# Efficacy of bedinvetmab on pain, activity, and body composition in obese dogs with osteoarthritis: A randomized double-blind placebo-controlled trial

**DOI:** 10.14202/vetworld.2026.1595-1610

**Published:** 2026-04-25

**Authors:** Aujchariyaporn Phongphuwanan, Sirinun Pisamai Tabtieang, Chutimon Thanaboonnipat, Teerapol Chinkangsadarn, Thita Taecholan, Kumpanart Soontornvipart

**Affiliations:** 1 Department of Veterinary Surgery, Faculty of Veterinary Science, Chulalongkorn University, Bangkok 10330, Thailand; 2 Nephrology, Cardiology, and Endocrinology Unit, Small Animal Teaching Hospital, Faculty of Veterinary Science, Chulalongkorn University, Bangkok 10330, Thailand

**Keywords:** accelerometer, activity monitoring, bedinvetmab, body composition, canine osteoarthritis, chronic pain, muscle preservation, obesity

## Abstract

**Background and Aim:**

Obesity and osteoarthritis (OA) frequently coexist in dogs and synergistically impair mobility, muscle integrity, and quality of life (QoL). Conventional management using nonsteroidal anti-inflammatory drugs may be limited by adverse effects, particularly in overweight and geriatric patients. Bedinvetmab, a canine anti–nerve growth factor monoclonal antibody, offers targeted analgesia with a favorable safety profile. This study aimed to evaluate the effects of bedinvetmab on daily physical activity, chronic pain, and body composition in obese dogs with OA.

**Materials and Methods:**

A 56-day prospective, randomized, double-blind, placebo-controlled clinical trial was conducted in 28 client-owned obese dogs diagnosed with OA. Dogs were allocated to receive either bedinvetmab (1 mg/kg) or placebo via subcutaneous injection on Days 0 and 28. Daily activity was quantified as the mean accelerometer-derived activity count per day (MAC/day). Pain and QoL were assessed using the Canine Brief Pain Inventory. Body composition parameters included body weight, body condition score, abdominal girth, thigh girth, and estimated body fat percentage. Data were analyzed using linear mixed-effects models, repeated-measures analysis of variance, and correlation analysis, with p < 0.05 considered significant.

**Results:**

The treatment group demonstrated significant improvements in both objective and subjective outcomes. MAC/day increased significantly over time, with a 25.6% rise from baseline by Day 56 (p = 0.032). Concurrently, total pain scores decreased significantly, with a mean reduction of 1.95 points (p < 0.001). A moderate negative correlation was observed between MAC/day and pain scores (r = −0.519, p < 0.001), indicating that reduced pain was associated with increased activity. Body weight did not change significantly; however, the treatment group exhibited a significant reduction in estimated body fat percentage and preservation of thigh muscle mass compared with placebo. No adverse effects were reported.

**Conclusion:**

Bedinvetmab is a safe and effective therapeutic option for managing OA-related pain in obese dogs, leading to improved mobility and QoL. Importantly, it contributes to favorable body composition changes, particularly muscle preservation, despite minimal weight loss. These findings support its integration into multimodal OA management strategies, especially in high-risk populations where conventional therapies are limited.

## INTRODUCTION

Obesity is a major health concern in dogs, with prevalence estimates of 26% [1]. This condition is multifactorial and contributes to a wide range of health risks, including osteoarthritis (OA), endocrinopathies, and cardiovascular disorders, paralleling similar concerns in human medicine [2, 3]. The relationship between obesity and OA is especially significant and contributes to a challenging cycle of pain and reduced mobility. This comorbidity represents a classic One Health challenge, as the cycle of pain and reduced mobility in dogs mirrors the clinical picture in human patients, where obesity is a leading risk factor for OA. Excessive calorie intake, inadequate physical activity, and genetic predisposition are recognized as key determinants of canine obesity [4]. Beyond the increased mechanical load placed on joints, obesity is now understood as a chronic inflammatory condition. Adipose tissue secretes pro-inflammatory cytokines, and hormones such as leptin can directly exacerbate cartilage damage and contribute to OA progression [5, 6]. This persistent pain leads to reduced activity, muscle weakness, and decreased energy expenditure, perpetuating both weight gain and joint deterioration.

Management of canine OA requires a comprehensive, multimodal therapeutic approach aimed primarily at improving quality of life (QoL). The cornerstone of management for OA in obese dogs is a dual approach: strategic weight management and pharmacological pain relief, most commonly with nonsteroidal anti-inflammatory drugs (NSAIDs). However, this standard of care presents significant challenges. Weight loss is often difficult to achieve due to low owner compliance and the dog’s pre-existing mobility limitations. Furthermore, long-term use of NSAIDs carries risks of gastrointestinal, renal, and hepatic adverse effects, which are of particular concern in older, overweight dogs that may have other underlying health issues. This creates a critical clinical gap for safer, more effective analgesic options in this specific, high-risk population.

Among the newer therapeutic options for chronic pain is the use of anti–nerve growth factor monoclonal antibodies (anti-NGF mAbs). These antibodies inhibit the interaction between nerve growth factor (NGF), a protein synthesized in response to noxious stimuli, and its receptors, including tropomyosin receptor kinase A and the low-affinity p75 neurotrophin receptor [7, 8]. This mechanism provides targeted analgesia with a prolonged half-life and potentially fewer systemic adverse effects compared with traditional medications [9], making it a promising alternative where NSAIDs are contraindicated or poorly tolerated.

Despite substantial advances in the management of OA, a critical gap remains in the evidence base concerning obese dogs, a population that represents a significant proportion of clinical cases. Most previous studies evaluating anti–NGF monoclonal antibodies, including bedinvetmab, have either excluded obese individuals or not stratified outcomes by body condition. This limitation is particularly important because obesity is not merely a mechanical burden but also a metabolically active state characterized by chronic low-grade inflammation, which may alter both disease progression and therapeutic response. Furthermore, current evaluations of treatment efficacy rely predominantly on subjective, owner-reported outcome measures, such as the Canine Brief Pain Inventory, which are inherently susceptible to bias, including placebo-by-proxy effects.

Another notable gap lies in the limited integration of objective functional assessments with morphometric or physiological indicators of body composition. Although increased physical activity is often assumed to correlate with improvements in muscle mass and reduction in adiposity, this relationship remains poorly defined in obese dogs with OA. In addition, there is a lack of studies examining whether targeted analgesic interventions can indirectly influence body composition parameters, such as muscle preservation and fat distribution, independent of weight loss. The absence of such multidimensional evaluations restricts a comprehensive understanding of therapeutic outcomes. Consequently, there is a need for well-designed randomized controlled trials that combine objective activity monitoring, validated pain scoring systems, and detailed body composition analysis to better characterize the clinical benefits of emerging therapies in this high-risk population.

Therefore, the primary aim of this study was to evaluate the efficacy of bedinvetmab in improving daily physical activity and reducing chronic pain in obese dogs with OA using a combination of objective and subjective outcome measures. Specifically, daily activity was quantified as the mean daily activity count derived from accelerometry, while pain and quality of life were assessed using the Canine Brief Pain Inventory.

As a secondary aim, the study sought to investigate the effects of bedinvetmab on body composition parameters, including body weight, body condition score, abdominal girth, thigh girth, and estimated body fat percentage. Additionally, the study aimed to examine the relationship between changes in physical activity and alterations in body composition to determine whether improvements in mobility are associated with measurable physiological benefits. By integrating objective digital biomarkers with clinical and morphometric assessments, this study was designed to provide a comprehensive evaluation of treatment response and to address existing gaps in the management of obese dogs with OA.

## MATERIALS AND METHODS

### Ethical approval

This study was conducted in accordance with internationally accepted guidelines for the care and use of animals in research. The experimental protocol was reviewed and approved by the Institutional Animal Care and Use Committee of Chulalongkorn University, Thailand (approval no.: #2431038). All procedures were performed in compliance with relevant national regulations governing animal experimentation and adhered to the principles outlined in the ARRIVE 2.0 guidelines.

Client-owned dogs diagnosed with OA were enrolled in the study with written informed consent obtained from their owners prior to participation. The study design followed a randomized, prospective, double-blind, placebo-controlled approach to ensure scientific rigor while minimizing bias. All interventions were performed by licensed veterinarians, and animal welfare was prioritized throughout the study period.

Bedinvetmab was administered at a clinically recommended dose (1 mg/kg, subcutaneously), and all animals were closely monitored for clinical response and potential adverse effects. No invasive or experimental procedures beyond standard veterinary clinical practice were performed. Pain management was considered an ethical priority; therefore, animals showing signs of severe discomfort or clinical deterioration were eligible for appropriate rescue therapy or withdrawal from the study.

All handling, monitoring, and data collection procedures were conducted to minimize stress and discomfort. The study also adhered to a One Health framework, recognizing the ethical responsibility to ensure animal welfare while generating clinically relevant data to improve the management of OA in companion animals.

### Study period and location

This study was conducted from August 2024 to April 2025 at the Surgery Unit of the Small Animal Teaching Hospital, Faculty of Veterinary Science, Chulalongkorn University, Bangkok, Thailand.

### Study design

The prospective, double-blind, randomized, placebo-controlled clinical trial was conducted. The sample size was determined using G*Power software (version 3.1.9.7). A *priori* power analysis was performed for a t-test comparing two independent means, using a significance level of α = 0.05 and statistical power of 0.80. The effect size was estimated from data reported in a prior proof-of-concept study on a feline-specific anti-NGF mAb on activity levels in cats [10], as this represented the most relevant available data at the time of study design. Specifically, Cohen’s d was calculated based on the mean ± standard deviation (SD) of the percentage change from baseline in activity counts for the placebo group (−5.3 ± 9.7) and the anti-NGF group (11.4 ± 21.6). This yielded a large anticipated effect size (Cohen’s d = 0.997). While the authors acknowledge the inherent limitations of extrapolating effect size across species, this approach was deemed a reasonable starting point given the conserved mechanism of NGF-mediated pain. Based on these parameters, a minimum of 28 dogs (14 per group) were required. To account for a potential dropout rate of approximately 10%, a total of 30 dogs were therefore enrolled to ensure the final sample size would provide adequate statistical power for the primary analysis.

To reflect real-world conditions and minimize disruption to the dogs’ daily routines, no modifications were made to the participants’ housing, diet, or exercise regimens. All dogs were privately owned and lived in household environments. Owners were explicitly instructed to maintain their dog’s existing dietary plan and normal physical activity levels throughout the study period.

### Animals

In total, 30 obese dogs with hindlimb mobility impairment due to OA were enrolled.

### Inclusion criteria

- Age of at least 1 year.
- A diagnosis of obesity, defined as a 9-point body condition score (BCS) of ≥7/9 [4].
- Evidence of mobility impairment in one or both hindlimbs associated with OA pain, confirmed by orthopedic examination and radiographic findings.
- A score of at least 2 out of 4 on the Canine OsteoArthritis Staging Tool (COAST) [11].

### Exclusion criteria

- Known or suspected neurological disease, severe systemic illness, chronic kidney disease (International Renal Interest Society stage ≥2), or significant cardiac disease.
- Endocrine disorders.
- Uncontrolled pruritic skin disease that could interfere with accurate activity measurements.
- Treatment with steroids, nonsteroidal anti-inflammatory drugs (NSAIDs), or anti-NGF mAb within 30 days prior to baseline. Concurrent use of dietary supplements was permitted if dosages remained unchanged throughout the study.

The dogs were stratified by COAST stage (2–4) and randomly assigned to one of two groups (n = 14 per group). Group A (control) received a placebo (saline solution), and Group B (intervention) received the canine-specific anti-NGF mAb bedinvetmab (Librela™; Zoetis Inc., Parsippany, NJ, USA). All treatments were administered via subcutaneous injection on days 0 and 28. The dose was calculated at 1 mg/kg based on actual body weight (BW), reflecting the drug’s dose-dependent nature. The study remained double-blinded for both the dog owners and the evaluator.

### Stratification and randomization

Subjects were stratified based on their COAST stage (2–4) and subsequently randomized into one of two groups: a placebo control group (n = 14) or an intervention group (n = 14). This stratification ensured a balanced distribution of disease severity between the groups. The random allocation sequence for each stratum was generated using a computer-based random number generator (RAND() function, Microsoft Excel) by a third party not involved in the clinical assessments. The control group received a placebo (saline solution), while the intervention group received the canine-specific anti-NGF mAb, bedinvetmab (Librela™; Zoetis Inc.).

### Intervention and blinding protocol

Treatments were administered via subcutaneous injection in the dorsal neck region on Day 0 and Day 28. The dosage was calculated at 1.0 mg/kg based on the dog’s actual BW. This dose, representing the upper limit of the manufacturer’s recommended range (0.5–1.0 mg/kg), was selected to maximize potential analgesic efficacy, reflecting the drug’s dose-dependent effect while remaining within a well-established safety margin [12, 13].

To preserve the integrity of the double-blind design, an unblinded veterinarian, who had no other role in the study, prepared the active drug and placebo in identical syringes. This protocol ensured that the investigators conducting assessments, the personnel administering the injections, and the dog owners remained blinded to group allocation throughout the 56-day study period.

### Study timeline

The study was conducted over 70 days. Baseline assessments were performed during the 14-day period prior to treatment. Subsequent evaluations took place on days 0, 28, and 56.

### Outcome measures

**Chronic pain and QoL assessment:** The canine brief pain inventory (CBPI) was used as the primary tool for owners to report their dogs’ pain levels and treatment response. The questionnaire has two sections, each scored from 1 to 10: the Pain severity score (CBPI PSS) and the CBPI pain interference scores (PIS). A 5-point Likert scale was also used to assess each dog’s QoL (1 = poor, 2 = fair, 3 = good, 4 = very good, 5 = excellent). Permission was obtained to translate the CBPI into Thai to facilitate communication with local owners.

**Daily physical activity assessment:** Daily physical activity was recorded using a collar-mounted accelerometer linked to the FitBark® device (FitBark Inc., Kansas City, MO, USA), connected to a smartphone application, which generated activity counts referred to as “Bark points.” Owners were instructed to ensure the device was worn 24 hours per day for the entire 70-day study period, as the battery life supported continuous monitoring. A day was considered valid for analysis if the device recorded data for a minimum of 20 hours. Weeks were included in the analysis if they contained at least 5 valid days. For weeks with missing daily data (e.g., due to temporary collar removal or transient signal loss) but meeting the 5-day minimum, the mean activity count per day (MAC/day) was calculated by averaging the data from the available valid days within that week.

Body composition assessment: Body composition parameters included BW, 9-point BCS, muscle condition score (MCS), abdominal girth (AG), and thigh girth (TG). BW was measured using the same digital scale throughout the study.

To minimize inter-observer variability, AG and TG were measured in triplicate by a single trained examiner using a Gulick II Tape Measure (Model 67020; Performance Health, Warrenville, IL, USA), and the mean of the three measurements was used for analysis. AG was taken at the level of the fifth to sixth lumbar vertebrae [14], while TG was measured at 70% of the thigh length (from the greater trochanter to the lateral fabella) with the dog in lateral recumbency and the leg extended [14].

The estimated percentage of body fat (%BF) was derived from AG using the formula: %BF = −12.937 + (0.696 × AG), as described by Laflamme [15].

To standardize the evaluation of progress while accounting for inaccuracies related to breed and size, all morphometric parameters were then used to calculate the relative percentage change per week (Δ%/wk), thereby focusing the analysis on intra-individual trends as determined by the following formulas:

Δ%BW/wk = (BW_current − BW_last) / BW_last × 100 / w

Δ%BF/wk = (BF_current − BF_last) / BF_last × 100 / w

BW_last = BW at previous visit, %BF_last = %BF at previous visit

BW_current = BW at current visit, %BF_current = %BF at current visit

w = number of weeks between visits

**Blood examination:** Blood samples were obtained at two time points: baseline (14 days prior to treatment) and on Day 56. All samples were collected following a minimum 12-hour fast. Samples were submitted to an accredited diagnostic laboratory (Veterinary Diagnostic Laboratory, Faculty of Veterinary Science, Chulalongkorn University) for a complete blood count and serum biochemistry profile, including alanine transaminase, alkaline phosphatase, blood urea nitrogen, creatinine, total protein, albumin, cholesterol, triglycerides, and thyroxine.

All results were interpreted in accordance with the laboratory-established reference intervals. These evaluations served to screen underlying systemic abnormalities prior to enrollment and to monitor for potential treatment-related adverse effects at the conclusion of the study.

### Statistical analysis

Statistical analyses were performed to assess within-group changes and between-group differences throughout the study. Categorical variables were summarized as counts and percentages. The distribution of all continuous variables was evaluated using the Shapiro–Wilk test, and results were reported as mean ± SD.

For within-group comparisons across time points, repeated-measures analysis of variance (ANOVA) was applied to parametric data, and the Friedman test was used for non-parametric data. Appropriate post hoc tests followed. Between-group comparisons were performed using linear mixed-effects models (LME) with fixed effects for group, time, and the group × time interaction. Model assumptions (normality of residuals and homogeneity of variance) were verified using residual plots.

LME inherently handles missing-at-random data, whereas a complete-case analysis was applied to all other tests. For single-time point comparisons between groups, independent-samples t-tests or Mann–Whitney U tests were used depending on the data distribution. Bonferroni correction was applied for multiple comparisons.

For all primary outcomes, effect sizes and their 95% confidence intervals (CIs) were calculated to assess the clinical relevance of the findings. Treatment response, defined as CBPI success or failure, was evaluated on days 28 and 56 according to established criteria [16]. Success was defined as a reduction of ≥1 point in CBPI PSS and ≥2 points in CBPI PIS from baseline. Fisher’s exact test was used to compare the number and percentage of responders in each group.

Correlation analyses were conducted using Pearson’s r for parametric variables and Spearman’s ρ for non-parametric variables. Correlation strength was classified as very weak (0.00–0.19), weak (0.20–0.39), moderate (0.40–0.59), strong (0.60–0.79), and very strong (0.80–1.00).

To minimize potential placebo-related bias, correlations involving pain-related scores were restricted to data from the treatment group. This analytical decision was made to specifically investigate the biological hypothesis of whether pain relief, as a direct result of bedinvetmab’s mechanism of action, translates into improved mobility. Including the placebo group could introduce confounding noise, as any observed correlations would be attributable to the placebo effect or random chance rather than the drug’s efficacy.

A p-value of <0.05 was considered statistically significant. All analyses were performed using SPSS Statistics v29.0.2.0 (IBM Corp.) and GraphPad Prism v10.3.0 (GraphPad Software).

## RESULTS

### Demographic characteristics

**Figure 1:**
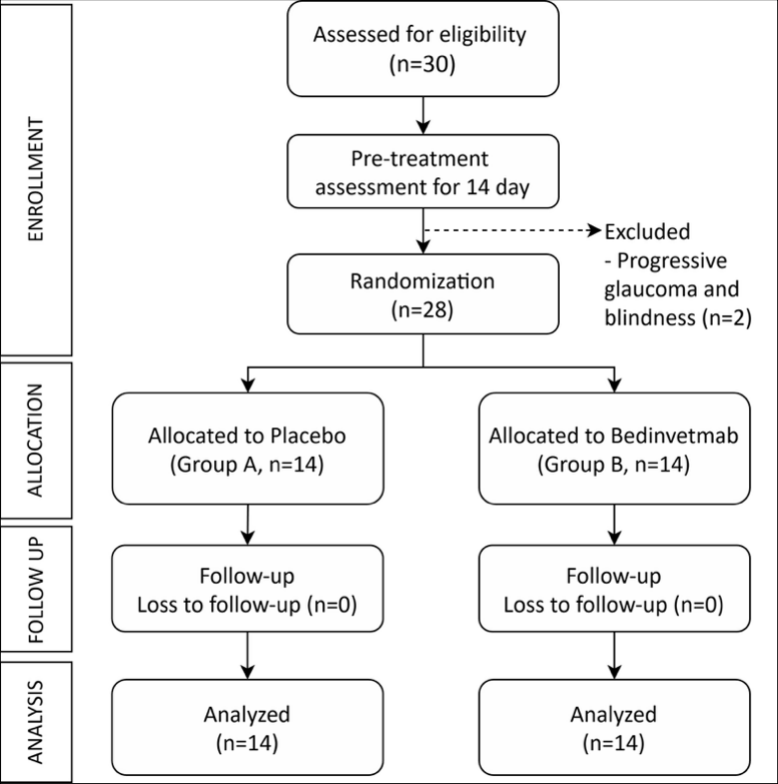
CONSORT flow diagram showing the progression of participants through enrollment, allocation, follow-up, and analysis phases of the study.

Thirty obese dogs with hindlimb mobility impairment due to OA were initially enrolled. All dogs underwent a comprehensive pre-treatment assessment. Two dogs were later excluded because of progressive glaucoma and blindness, conditions that would have interfered with the accurate evaluation of daily physical activity. The final analysis, therefore, included 28 dogs: 13 females (46.4%) and 15 males (53.6%), with a mean age of 9.75 ± 3.47 years (range: 2–16 years). Most dogs were neutered (n = 25, 89.3%). The flow of participants through each stage of the trial is detailed in Figure 1.

The breed distribution was diverse. Pomeranians were the most common (n = 7, 25.0%), followed by chihuahuas (n = 4, 14.3%), mixed-breed dogs (n = 4, 14.3%), and poodles (n = 3, 10.7%). Other represented breeds included Shih Tzus, bulldogs, Finnish spitz, French bulldogs, golden retrievers, Labrador retrievers, Samoyeds, Welsh corgis, and Yorkshire terriers.

The hip joint was the most frequently affected, with all 28 dogs (100%) showing bilateral hip involvement. Concurrent stifle joint involvement was present in eight dogs (28.6%). At enrollment, BCS values were distributed as follows: 7/9 (n = 11, 39.3%), 8/9 (n = 14, 50.0%), and 9/9 (n = 3, 10.7%). The MCS was normal in most dogs (n = 19, 67.9%), with mild (n = 7, 25.0%) and moderate (n = 2, 7.1%) muscle loss. Mean BW was 12.88 ± 12.35 kg (range: 3–47 kg).

Based on pre-treatment assessments, the dogs were assigned COAST stage 2 (n = 9), stage 3 (n = 10), or stage 4 (n = 9), and then randomly allocated to Group A (placebo, n = 14) or Group B (treatment, n = 14).

Comparison of baseline characteristics prior to intervention (Table 1) showed no statistically significant differences between groups in age, BW, BCS, COAST stage, %BF, AG, left TG (LTG), right TG (RTG), MAC/day, CBPI scores, CBPI PSS, CBPI PIS, CBPI QoL, serum cholesterol, or serum triglycerides (all p > 0.05). Thus, Groups A and B were statistically comparable across all measured parameters at baseline.

**Table 1:** Baseline characteristics of dogs in Group A (placebo) and Group B (treatment). Data are presented as mean ± SD or median (range).

Parameter	Group A (n = 14)	Group B (n = 14)	p-value
Age (years)	9.86 ± 3.18	9.64 ± 3.86	0.874^a^
BW (kg)	8.47 ± 6.00	8.95 ± 15.40	0.232^b^
CBPI	5.16 ± 2.06	4.56 ± 1.27	0.363^a^
CBPI PSS	2.42 ± 1.60	2.59 ± 0.86	0.582^a^
CBPI PIS	2.81 ± 1.21	1.90 ± 0.77	0.197^a^
AG (cm)	45.89 ± 9.00	57.59 ± 17.76	0.066^b^
Left TG (cm)	21.56 ± 8.37	21.94 ± 8.80	0.818^b^
Right TG (cm)	21.72 ± 8.75	22.03 ± 8.94	0.581^b^
MAC/day (points/day)	3015.29 ± 762.23	2719.64 ± 809.34	0.329^a^
CBPI QoL (1–5)	2 (1–3)	2 (1–3)	0.112^b^
BCS (9-point)	8 (7–9)	8 (7–9)	0.635^b^
MCS (0–3)	0 (0–2)	0 (0–2)	0.716^b^
COAST stage (2–4)	3 (2–4)	3 (2–4)	0.643^b^

a Independent-samples t-test, bMann–Whitney U test. BW = body weight, CBPI = Canine brief pain inventory, PSS = Pain severity score, PIS = Pain interference score, AG = Abdominal girth, TG = Thigh girth, MAC/day = Mean activity count per day, QoL = Quality of life, BCS = Body condition score, MCS = Muscle condition score, COAST = Canine osteoarthritis staging tool.

No adverse effects were observed in any dogs during the study following subcutaneous administration of either bedinvetmab or normal saline.

### Daily physical activity

**Figure 2:**
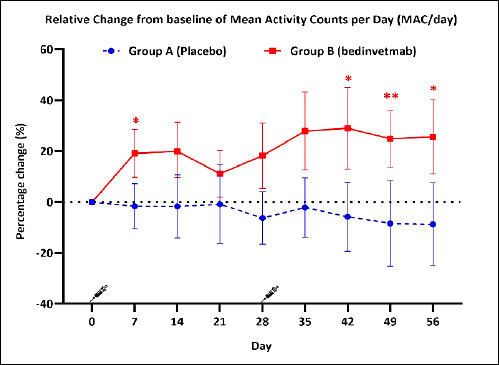
Percentage change from baseline in mean activity count per day (MAC/day) from Day 0 to Day 56 in dogs receiving placebo (Group A; blue circles, dashed line) or bedinvetmab 1 mg/kg subcutaneously (Group B; red squares, solid line). Treatments were administered on Days 0 and 28. Data are presented as mean percentage change (%) with 95% confidence intervals. Statistically significant within-group changes from baseline: *p < 0.05, **p < 0.01.

Within-group comparisons of MAC/day were analyzed using repeated-measures ANOVA (Table 2). In Group A, no significant change in MAC/day was observed across the study period (p = 0.380), and no pairwise time points differed significantly from baseline. By contrast, Group B showed a significant change over time (p < 0.001). Post hoc pairwise comparisons in Group B revealed a significant increase in MAC/day compared with baseline. Specifically, there was a 19.15% rise on Day 7 (p = 0.023), followed by 19.93% on Day 14 (p = 0.103). Although a slight decline to 11.14% was observed on Day 21 (p = 0.765), the upward trend resumed from Day 28 onward. Notably, a sustained and statistically significant increase was seen on Day 42 (29.00%, p = 0.007) and Day 56 (25.60%, p = 0.032) (Figure 2).

**Table 2:** Percentage of relative changes from baseline in mean activity count per day (MAC/day) in Group A (placebo) and Group B (bedinvetmab). Data are presented as mean percentage change (%) with 95% CI.

Group	Day 7	Day 14	Day 21	Day 28	Day 35	Day 42	Day 49	Day 56
A	−1.70 [−10.55, 7.15]	−1.74 [−14.12, 10.63]	−0.89 [−16.34, 14.57]	−6.25 [−16.58, 4.07]	−2.14 [−13.83, 9.55]	−5.80 [−19.32, 7.72]	−8.44 [−25.26, 8.38]	−8.78 [−25.13, 7.57]
B	19.15^†††^ [9.75, 28.54]	19.93 [9.71, 31.35]	11.14 [1.95, 20.33]	18.20 [5.40, 31.11]	27.89 [12.59, 43.20]	29.00 [12.97, 45.03]	24.85 [13.66, 36.05]	25.60 [10.98, 40.21]
p-value^a^	0.023*	0.103	0.765	0.152	0.076	0.047*	0.007**	0.032*

Within-group comparison by repeated-measures ANOVA: †††p < 0.001. Post hoc paired t-tests for within-group comparisons:

* p < 0.05,

** p < 0.01. ap-values refer to between-group comparisons.

Between-group comparisons using LME showed a significant main effect of group (p < 0.001, η²p = 0.380), but no significant effects for time (p = 0.572, η²p = 0.036) or the group × time interaction (p = 0.434, η²p = 0.043) (Table 3). The lack of a significant group × time interaction suggests that the pattern of activity change over the study period followed parallel trajectories in both groups.

**Table 3:** Linear mixed-effects model analysis of Canine Brief Pain Inventory (CBPI) scores and subdomains (Pain Severity Score and Pain Interference Score) at Day 28 and Day 56. Data are reported as mean differences with 95% confidence interval (CI).

Parameter	Group effect p (η²p)	Time effect p (η²p)	Group × time p (η²p)	Time point	Mean difference (95% CI)	p-value
MAC/day	<0.001*** (0.380)	0.572 (0.036)	0.434 (0.043)	–	–	–
CBPI	0.027* (0.163)	<0.001*** (0.319)	0.011* (0.146)	Day 28	−0.74 [−1.16, −0.31]	<0.001^†††^
				Day 56	−1.22 [−1.82, −0.61]	<0.001^†††^
CBPI PSS	0.12 (0.221)	0.003** (0.197)	0.021* (0.139)	Day 56	−0.44 [−0.75, −0.13]	0.003^††^
CBPI PIS	0.115 (0.093)	<0.001*** (0.357)	0.115 (0.080)	Day 14	−0.47 [−0.74, −0.20]	0.005^††^
				Day 56	−0.75 [−1.06, −0.44]	<0.001^†††^

Type III tests of fixed effects:

* p < 0.05,

** p < 0.01,

*** p < 0.001. Pairwise comparisons: †††p < 0.001, ††p < 0.01. CBPI = Canine brief pain inventory, PSS = Pain severity score, PIS = Pain interference score, MAC/day = Mean activity count per day.

### Chronic pain and QoL

The proportion of dogs meeting the predefined criteria for treatment response was consistently higher in Group B than in Group A on both Day 28 and Day 56 (Table 4). By Day 28, 64.28% of dogs in Group B were classified as responders compared with a substantial 50.00% in Group A (p = 0.704). The response rate in Group B increased further by Day 56, with 85.71% of dogs in Group B achieving treatment success compared with a constant 50.00% in Group A (p = 0.103).

**Table 4:** Treatment response based on Canine brief pain inventory criteria on Day 28 and Day 56. Proportion of dogs (%) meeting predefined response criteria (≥1 reduction in CBPI PSS and ≥2 reduction in CBPI PIS). Comparisons performed using Fisher’s exact test.

Time	Outcome	Group A (n = 14)	Group B (n = 14)	p-value
Day 28	Success	7 (50.0%)	9 (64.28%)	0.704
	Failure	7 (50.0%)	5 (35.71%)	–
Day 56	Success	7 (50.0%)	12 (85.71%)	0.103
	Failure	7 (50.0%)	2 (14.29%)	–

CBPI = Canine Brief Pain Inventory; PSS = Pain Severity Score; PIS = Pain Interference Score.

Within-group changes over the study period were assessed using repeated-measures ANOVA (Table 5). Group B showed significant changes in the total CBPI score (p < 0.001), with reductions from baseline observed on both Day 28 (−1.00, 95% CI: −3.2 to 0.8, p = 0.003) and Day 56 (−1.95, 95% CI: −3.8 to −0.7, p < 0.001). Similarly, significant reductions were evident in the CBPI PIS, with changes of −0.50 (95% CI: −1.70 to 0.20, p = 0.002) on Day 28 and −1.20 (95% CI: −1.8 to 1.0, p = 0.001) on Day 56. The CBPI PSS also demonstrated improvement by Day 56 (−0.90, 95% CI: −2.1 to 0.4, p = 0.002), although the change on Day 28 was not statistically significant (p = 0.080). By contrast, no significant within-group changes were observed in any CBPI component in Group A during the study period (all p > 0.05).

**Table 5:** Median change in Canine brief pain inventory scores (points) from baseline on Day 28 and Day 56 in Group A (placebo) and Group B (bedinvetmab). Data are presented as median change (range). CBPI (max score: 10), CBPI PSS (max: 4), CBPI PIS (max: 6).

Score	Group	Day 28	Day 56
CBPI	A	−0.25 (−1.60 – 0.60)	−0.30 (−2.80 – 2.70)
	B	−1.00 (−3.20 – 0.80)^†††^	−1.95 (−3.80 – −0.70)^†††^
CBPI PSS	A	−0.50 (−1.10 – 0.80)	−0.50 (−1.50 – 1.60)
	B	−0.50 (−1.80 – 0.60)	−0.90 (−2.10 – 0.40)
CBPI PIS	A	−0.25 (−1.80 – 1.30)	−0.50 (−1.80 – 1.20)
	B	−0.50 (−1.70 – 0.20)	−1.20 (−1.80 – 1.00)

Within-group comparison by repeated-measures ANOVA: †††p < 0.001. p-values in Group B refer to paired t-tests vs baseline: **p < 0.01, ***p < 0.001. CBPI = Canine brief pain inventory, PSS = Pain severity score, PIS = Pain interference score.

Between-group comparisons (Table 3) for the total CBPI score revealed significant main effects of group (p = 0.027, η²p = 0.163), time (p < 0.001, η²p = 0.319), and the group × time interaction (p = 0.011, η²p = 0.146). Pairwise comparisons showed significant reductions from baseline at Day 28 (−0.74, 95% CI: −1.16 to −0.31, p < 0.001) and Day 56 (−1.22, 95% CI: −1.82 to −0.61, p < 0.001). For the CBPI PSS, a significant time effect (p = 0.003, η²p = 0.197) and group × time interaction (*p* = 0.021, η²p = 0.139) were observed, with a significant reduction from baseline detected on Day 56 (−0.44, 95% CI: −0.75 to −0.13, p = 0.003). The CBPI PIS showed a significant time effect (p < 0.001, η²p = 0.357), with reductions on Day 14 (−0.47, 95% CI: −0.74 to −0.20, p = 0.005) and Day 56 (−0.75, 95% CI: −1.06 to −0.44, p < 0.001), although neither the group effect nor the group × time interaction reached statistical significance.

For CBPI QoL (Table 6), within-group analysis revealed significant improvement in Group B at both Day 28 (p = 0.024) and Day 56 (p = 0.004). No significant changes were observed in Group A at either time point (both p > 0.025). However, between-group comparisons on Day 28 and Day 56 did not show statistically significant differences between groups (all p > 0.025).

**Table 6:** Percentage changes per week in BW, morphometrics, BCS, MCS, and QoL. Data for %BW, AG, %BF, and TG are presented as mean ± SEM for relative percentage change per week (Δ%/wk). BCS, MCS, and CBPI QoL are presented as median (range).

Parameter	Group	Day 28	p-value^b^	Day 56	p-value^b^	Friedman p-value^a^	Between-group p-value^c^ (Day 28 / Day 56)
%BW	A	0.29 ± 0.17%	0.279	0.27 ± 0.09%	0.331	0.191	0.734 / 0.011^‡^
	B	0.24 ± 0.12%	0.929	−0.06 ± 0.08%	0.248	0.064	–
AG	A	1.79 ± 0.73%	0.018*	2.41 ± 0.92%	0.027	0.001^††^	0.454 / 0.874
	B	−3.18 ± 0.98%	<0.001***	−6.15 ± 1.10%	<0.001***	<0.001^†††^	<0.001^†††^
%BF	A	0.74 ± 0.29%	0.463	0.21 ± 0.60%	0.925	0.318	<0.001^‡‡‡^ / 0.005^‡‡^
	B	−1.53 ± 0.36%	0.041	−1.65 ± 0.61%	0.124	0.072	–
Left TG	A	−0.24 ± 0.09%	0.028	−0.36 ± 0.09%	0.001**	<0.001^†††^	0.581 / 0.323
	B	1.23 ± 0.47%	0.023*	0.90 ± 0.32%	0.033	0.423	–
Right TG	A	−0.01 ± 0.57%	0.028	−0.14 ± 0.27%	0.008**	<0.001^†††^	0.370 / 0.260
	B	0.96 ± 0.52%	0.148	0.70 ± 0.37%	0.124	0.383	–
BCS (9-point)	A	8 (7–8)	1.000	8 (7–8)	1.000	1.000	0.511 / 0.511
	B	8 (7–9)	1.000	8 (6–9)	0.014*	0.002^††^	–
MCS	A	0 (0–2)	1.000	0 (0–2)	1.000	1.000	0.716 / 0.716
	B	0 (0–2)	1.000	0 (0–2)	1.000	1.000	–
CBPI QoL	A	2 (2–5)	0.527	3 (1–4)	0.366	0.682	0.769 / 0.246
	B	2.5 (1–4)	0.024*	3 (2–5)	0.004**	<0.001^†††^	–

Within-group: aFriedman test (†p < 0.05, ††p < 0.01, †††p < 0.001); bWilcoxon signed-rank test (*p < 0.025, **p < 0.01, ***p < 0.001). Between-group: cMann–Whitney U test (‡p < 0.025, ‡‡p < 0.01, ‡‡‡p < 0.001). Significance threshold adjusted to p < 0.025. %BW = percentage change in body weight, AG = Abdominal girth, %BF = Estimated body fat percentage, TG = Thigh girth, BCS = Body condition score, MCS = Muscle condition score, CBPI QoL = Canine brief pain inventory quality of life.

### Body composition

Within-group comparisons using the Friedman test (Table 6) showed significant differences in Δ%/wk of AG, LTG, and RTG in Group A (*p* = 0.001, p < 0.001, and p < 0.001, respectively). In Group B, significant changes were found in AG (p < 0.001) and BCS (p = 0.002). Post hoc analysis with the Wilcoxon signed-rank test clarified these findings. By Day 28, AG in Group A had increased significantly (1.79% ± 0.73, p = 0.018), whereas Group B showed a marked reduction (−3.18% ± 0.98, p < 0.001). By Day 56, Group A exhibited a significant reduction in TG (LTG: −0.36% ± 0.09, p < 0.001; RTG: −0.14% ± 0.27, p = 0.008). By contrast, Group B maintained stable TG while continuing to show a reduction in AG (−6.15% ± 1.10 per week, p < 0.001).

Between-group comparisons revealed statistically significant differences in the relative change of %BW and the estimated %BF. A significant difference in %BW was observed on Day 56 (p = 0.011), while the change in estimated %BF differed significantly at both Day 28 (p < 0.001) and Day 56 (p = 0.005). On Day 28, Group B demonstrated an estimated weekly %BF loss of 1.53% ± 0.36% per week, whereas Group A showed an estimated gain of 0.74% ± 0.29% per week. This pattern continued through Day 56, with Group B maintaining an estimated weekly %BF loss of 1.65% ± 0.61% per week, while Group A showed a minimal gain from baseline (0.21% ± 0.60% per week).

### Correlation analysis

**Figure 3:**
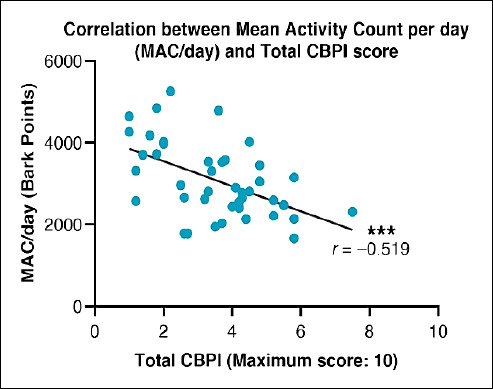
Correlation between mean activity count per day (MAC/day; Bark points) and total Canine brief pain inventory (CBPI) score. Each data point represents an individual assessment. The solid line shows the linear regression fit. A moderate negative correlation was observed (Pearson r = −0.519, p < 0.001).

A moderate negative correlation was observed between MAC/day and the overall CBPI score (r = −0.519, p < 0.001) (Figure 3), with notable correlations also found in the PIS (r = −0.443, p = 0.003) and QoL subdomains (ρ = −0.456, p = 0.002). Within the PSS, a weak but statistically significant correlation was identified (r = −0.377, p = 0.014). QoL was also negatively correlated with the total CBPI score (ρ = −0.346, p = 0.025), with the strongest association again observed in the PIS subdomain (ρ = −0.464, p = 0.002). No statistically significant correlations were found between MAC/day and any body composition parameters (all p ≥ 0.05).

## DISCUSSION

The findings of this study are particularly significant because they provide the first evidence from a randomized, prospective, double-blind, placebo-controlled trial evaluating bedinvetmab specifically in obese dogs with OA. This work directly addresses a previously underrepresented population in anti-NGF therapy research and offers new insights into the management of the common comorbidity of obesity and OA.

### Demographic and baseline characteristics

The study cohort was predominantly composed of older, neutered dogs, with a mean age of 9.75 ± 3.47 years (range: 2-16 years). This finding is consistent with the established epidemiology of OA as a degenerative joint disease whose prevalence increases with advancing age [17]. Because bedinvetmab is contraindicated in growing animals, dogs younger than 1 year were excluded.

A high proportion of enrolled dogs were neutered (89.3%), which is consistent with previous reports identifying neutering as a significant risk factor for postoperative weight gain, elevated BCS, and increased risk of orthopedic disorders [18, 19]. Gonadectomy has been associated with metabolic alterations, underscoring the need for preventive strategies to control weight gain after neutering [20, 21].

Sex distribution was nearly equal (53.6% male, 46.4% female). Previous studies suggest that female dogs may be more prone to obesity [22], whereas males may exhibit a greater predisposition to OA [23], potentially reflecting hormonal and lifestyle influences on these conditions.

In terms of breed representation, toy breeds were disproportionately represented, particularly Pomeranians, Chihuahuas, and mixed-breed dogs. This differs from earlier studies reporting a greater risk of obesity in larger breeds, such as retrievers and spaniels [18, 19]. The present findings may reflect regional breed demographics, although emerging evidence suggests that toy breeds may also have a 30%-50% increased risk of becoming overweight [22].

### Effect on daily physical activity, chronic pain score, and QoL

Significant increases in MAC/day were observed in the bedinvetmab-treated group. Improvements emerged as early as Day 7, with a 19.15% increase, peaked around Day 14, and remained elevated at 11.14% by the third week. These improvements are comparable to those reported previously [10, 24]. A transient decline was noted thereafter, followed by a further rise through Day 56, shown in Figure2. The observed activity pattern aligns with the established pharmacokinetic and pharmacodynamic profiles of canine anti-NGF mAb therapy, which indicate therapeutic onset by Day 7, maximum plasma concentrations at approximately 5-7 days, a half-life of approximately 10 days, and steady-state levels after two doses [9, 10, 25].

Between-group comparisons showed a significant main effect of group (p < 0.001), indicating a consistent difference in daily physical activity between the treatment and placebo groups throughout the study period. However, neither a significant effect of time nor a group × time interaction was observed, suggesting parallel trends over time. Within-group analysis further supported a sustained upward trajectory in the treatment group, particularly after the second administration of bedinvetmab. The observed plateau likely reflects therapeutic maintenance after steady-state is reached, allowing continued analgesic benefit without further peak effects. These findings suggest that, although clinical improvement is detectable within the first week after treatment, a second dose is essential to achieve and sustain therapeutic consistency. This is consistent with long-term OA management, which often requires treatment durations beyond 28 days [26].

Accelerometer-based monitoring with FitBark®, a commercially available wearable device, provided an objective method for evaluating physical activity. Previous studies support the reliability of FitBark® in dogs, with strong correlations documented against step counts during off-leash activity [27] and against Actical® accelerometers during week-long monitoring periods [28]. Although real-time feedback may enhance owner engagement, it may also introduce motivational bias, whereby owners may have subconsciously encouraged more activity, potentially influencing the study outcomes. This factor should be considered when interpreting the activity data and represents a limitation that future studies could reduce by blinding owners to device feedback. In addition, these findings have One Health implications by positioning the tracker as a digital biomarker with translational relevance. The motivational effect on owners suggests that improving a dog’s mobility may promote a more active lifestyle for both pet and owner, thereby strengthening the human-animal bond.

Owner-reported outcomes assessed using the CBPI also demonstrated marked improvement in the treatment group. Total CBPI scores decreased significantly from baseline by Day 28, consistent with previous findings reporting maximal effect around Day 42, although improvements may occur as early as Day 7 [29]. In this study, dogs receiving bedinvetmab showed substantial reductions in pain scores, with mean total CBPI reductions of approximately 2 out of 10 points (range: 0.7–3.8) from baseline by Day 56. PSS decreased by 0.9 out of 4 (range: –0.4 to 2.1), and PIS decreased by 1.2 out of 6 (range: –1.8 to 1.0), alongside a 25.6% increase in MAC/day. Between-group comparisons further confirmed significant differences in total CBPI scores at both Day 28 and Day 56, reinforcing the superior analgesic effect of bedinvetmab compared with placebo. Moreover, correlation analysis revealed significant negative associations between MAC/day and total CBPI score (r = –0.519, p < 0.001), CBPI PIS (r = –0.443, p = 0.003), and CBPI PSS (r = –0.377, p = 0.014), indicating that as pain decreased, physical activity increased.

Treatment success was achieved in 64.28% and 85.71% of dogs in the treatment group by days 28 and 56, respectively, compared with a constant 50.00% in the placebo group. These results are in agreement with previous studies reporting lower placebo success rates [29], suggesting a potential placebo-by-proxy effect in the double-blinded design, whereby owner expectations may influence assessment of the pet’s condition. Nevertheless, the significantly greater and progressive improvement in the bedinvetmab group on both objective (MAC/day) and subjective (CBPI) measures provides strong evidence of the drug’s true analgesic effect beyond this placebo response.

In terms of QoL, owner-reported ratings improved substantially in the treatment group, with scores increasing from fair or good to very good or excellent by Day 56. Statistically significant improvements were observed by Day 28 (*p* = 0.024) and again by Day 56 (*p* = 0.004), in agreement with previous studies [7, 29]. In contrast, QoL scores in the placebo group remained unchanged. Additionally, a significant negative correlation was found between QoL and CBPI, particularly the PIS subdomain (ρ = -0.464, p = 0.002). These findings suggest that, as pain interference decreases, owner-perceived QoL improves. Taken together, the improvements in both pain and QoL reinforce the importance of chronic pain control in the management of OA-associated pain and in enhancing patient QoL.

### Effect on body composition

**Figure 4:**
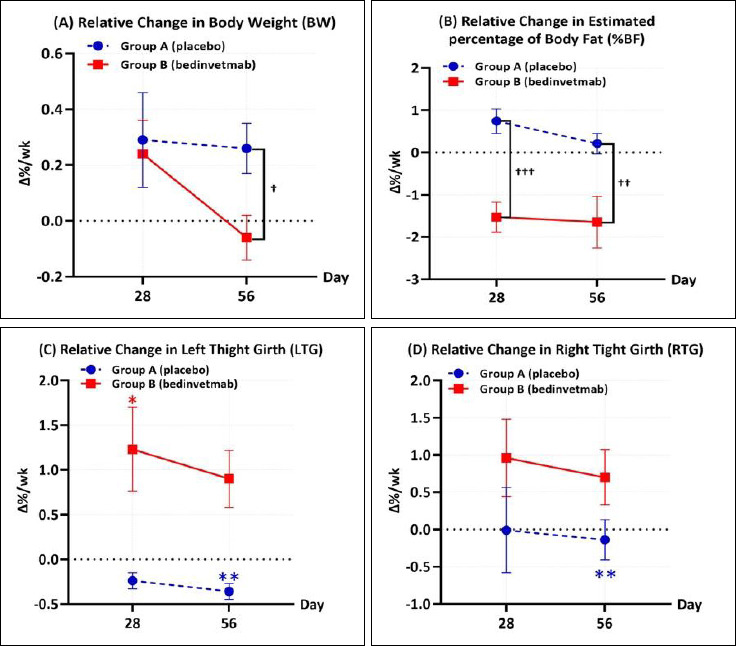
Relative percentage change per week (Δ%/wk) in body composition parameters for dogs treated with placebo (Group A; blue circles, dashed line) or bedinvetmab 1 mg/kg subcutaneously (Group B; red squares, solid line). Graphs show data at Days 28 and 56 for (A) body weight, (B) estimated body fat percentage, (C) left thigh girth, and (D) right thigh girth. Asterisks indicate significant within-group changes from baseline (Wilcoxon signed-rank test: *p < 0.025, **p < 0.01). Daggers indicate significant between-group differences (Mann–Whitney U test: †p < 0.025, ††p < 0.01, †††p < 0.001).

In the present study, Δ%/wk of AG, BW, BCS, %BW, and %BF were used to assess changes in body composition. The treatment group showed significant differences in Δ%/wk of %BW and %BF (Figures 4A and 4B), with a mean %BW change of -0.06% per week. Although this rate was below the recommended clinical target of 1%-2% reduction per week [30], it remained clinically meaningful when compared with the unfavorable trajectory observed in the placebo group. Weight reduction typically requires a multimodal approach that includes diet, exercise, and owner education, especially in geriatric or neutered dogs with reduced energy expenditure [4]. The observed weekly %BF reduction of 1.6%, estimated morphometrically, is slightly greater than prior reports of 0.57%–1.43% obtained using dual-energy X-ray absorptiometry [31, 32]. However, to the authors’ knowledge, no established recommendations define the optimal weekly rate of %BF reduction in veterinary clinical practice.

A divergence in TG trends was observed between groups. By Day 56, TG decreased in the placebo group at a rate of 0.14% per week for the right thigh and 0.36% per week for the left thigh. In contrast, the treatment group showed slight increases of 0.70% and 0.90% per week for the right and left thighs, respectively (Figures 4C and 4D), suggesting preservation of muscle mass. Maintenance of thigh muscle mass is essential for supporting mobility, particularly in dogs with OA [33].

Various morphometric approaches have been proposed for assessing body composition in dogs, including AG with or without body-length ratios and body mass index calculations [34, 35]. Waist circumference is widely recognized in human medicine as a surrogate marker of central obesity, which is associated with visceral fat and increased risk of various disorders, including OA [36]. In veterinary medicine, AG-based indices have been correlated with intra-abdominal fat and increased cardiac risk [37]. However, the AG-based body fat estimation formula proposed by Laflamme [15] may have limited applicability because of breed-related variations in body conformation, analogous to ethnicity-related differences in body composition observed in humans [15, 38]. Therefore, the calculated %BF values should be interpreted as indicators of relative change and divergent group trends rather than as definitive absolute percentages. Despite these limitations, such assessments remain clinically valuable, particularly when advanced tools are unavailable. Nonetheless, the absence of standardized guidelines highlights the need for improved methodologies. Ultrasonography may offer a more reliable alternative for evaluating changes in muscle and fat, while reducing variability associated with coat clipping, tape tension, or positioning [15, 39].

Importantly, owner perception plays a crucial role in the management of OA and obesity. Evidence indicates that many owners underestimate their dog’s body condition and overestimate activity levels, with only 13% accurately assessing body condition in recent surveys [40–42]. Such misperception may delay recognition and intervention.

### Adverse effects

No adverse effects were observed following bedinvetmab administration, supporting its favorable safety profile when administered at 1 mg/kg subcutaneously on a monthly basis. Unlike NSAIDs, which act through cyclooxygenase inhibition and are associated with gastrointestinal and renal adverse effects, bedinvetmab exerts its analgesic effect through NGF neutralization, offering a safer alternative for long-term use in dogs with chronic OA [43]. Its wide safety margin [25] is particularly advantageous in cases in which NSAID use is contraindicated. In addition, bedinvetmab reduces concerns regarding overdose and dose-dependent adverse effects often encountered with NSAIDs in overweight individuals.

### Interpretation

This study confirms our hypothesis that monthly administration of bedinvetmab significantly improves mobility and pain management in obese dogs with OA. Treated dogs showed marked improvement in both MAC/day and CBPI scores compared with the placebo group. The observed negative correlation between these two metrics further suggests that bedinvetmab effectively alleviates OA-related chronic pain, leading to enhanced mobility and improved QoL.

Regarding body composition, bedinvetmab treatment resulted in favorable changes, proving effective in reducing both BCS and AG, with a statistically significant relative reduction in estimated %BF compared with the placebo group. Importantly, the treatment was also effective in preserving thigh muscle mass, as reflected by stable TG values in the treatment group, in contrast to the reductions observed in the placebo group. Muscle preservation is crucial in OA management because it contributes to joint stability and functional recovery. These findings add to the existing literature by demonstrating that bedinvetmab improves objective mobility and preserves muscle mass in obese dogs with OA. By alleviating pain and improving mobility, the treatment may help counteract muscle atrophy, a common consequence of pain-induced lameness [44], which often requires interventions such as physiotherapy to reverse [45]. This pattern is consistent with the findings of Soder *et al*. [46], who reported similar positive changes, including decreased BCS and AG and improved thigh circumference, after 56 days of reinforced home-based exercise, despite no significant change in BW.

Contrary to our secondary hypothesis, no statistically significant correlation was found between changes in activity levels and improvements in body composition parameters. This finding suggests that the positive changes in body composition following bedinvetmab treatment may be mediated by factors beyond increased physical activity, highlighting the complexity of canine obesity management.

Although this study did not demonstrate a direct correlation, the broader literature consistently supports an association between higher physical activity levels and improved body condition in dogs [47]. While activity alone is often insufficient to achieve clinically meaningful weight loss, it remains a critical component of obesity management because of its wide-ranging benefits, including muscle preservation, BW regulation, cardiovascular health, and improved socialization [48].

### Limitations of the study

This study has several limitations that warrant consideration. A primary limitation was the absence of strict dietary standardization and monitoring, which introduced a potentially important confounding factor for body composition outcomes. Although owners were instructed to maintain their dogs’ usual routines, unrecorded variations in caloric intake or exercise may have influenced the observed results. Furthermore, the assessment of mobility was constrained by the lack of objective, granular gait analysis tools, such as force plates or kinematic assessments. Such instrumentation would have provided specific biomechanical data on limb function and lameness, yielding more direct quantification of OA-associated pain. In addition, the study did not include analysis of metabolic markers, such as inflammatory cytokines or metabolic hormones. Inclusion of these markers would have strengthened the study by providing mechanistic insight into the observed body composition changes.

Another methodological limitation relates to the *a priori* sample size calculation, which was based on an effect size derived from a study of a feline-specific anti-NGF mAb in cats. Although this represented the best available evidence at the time of study design, this cross-species extrapolation should be acknowledged. Physiological and metabolic differences between species may mean that the true effect size in dogs differs from our estimate, potentially affecting the statistical power of the study. Furthermore, the predominance of small and toy breeds inherently limits the direct generalizability of our findings because of possible differences in biomechanics, pathophysiology, and drug metabolism. Therefore, these results should be extrapolated to large-breed dogs with caution.

The CBPI used in this study was a Thai translation that, although prepared according to standard procedures, has not undergone formal validation. While it represented the best available instrument for the study population, nuances lost in translation may have influenced the owner-reported scores.

Finally, the follow-up period of 8 weeks may have been insufficient to assess long-term changes in body composition or the durability of analgesic effects. Ethical constraints related to withholding effective pain relief limit the feasibility of an extended placebo-controlled design. Because weight loss is inherently gradual, future studies spanning 6-12 months without a control group may be better suited to evaluate the long-term impact of bedinvetmab.

### Future research directions

Future studies should investigate the long-term effects of anti-NGF mAb therapy and its integration into multimodal OA management. Such an approach should include dietary control, physical rehabilitation, and anti-NGF mAb administration, with the aim of optimizing clinical outcomes, including pain relief, weight reduction, and improved mobility. A logical next step would be a prospective, randomized controlled trial with an extended follow-up duration of 6-12 months. This timeframe would provide a more realistic window for assessing meaningful changes in body composition and establishing long-term safety profiles. Furthermore, future trials would be methodologically strengthened by the inclusion of objective gait analysis, such as force plate or kinematic assessments, to provide quantitative measures of functional improvement. Although the present study provides a strong foundation, further research is needed to support broader clinical application and to evaluate its relevance across other species with comparable conditions.

## CONCLUSION

This randomized, prospective, double-blind, placebo-controlled study demonstrates that monthly administration of bedinvetmab provides significant clinical benefits in obese dogs with OA. Treatment resulted in marked improvements in objective physical activity (MAC/day), alongside significant reductions in pain severity and interference as measured by CBPI, with effects evident as early as Day 7 and sustained through Day 56. Importantly, these improvements were accompanied by enhanced owner-perceived QoL and a strong negative correlation between activity and pain scores, confirming that analgesic efficacy translated into meaningful functional outcomes.

In addition to mobility and pain relief, bedinvetmab treatment was associated with favorable changes in body composition, including reductions in BCS, AG, and estimated %BF, as well as preservation of thigh muscle mass compared with the placebo group. These findings highlight a clinically relevant secondary benefit, whereby effective pain control may mitigate disuse-related muscle loss and support functional recovery in dogs with OA. The absence of adverse effects further supports the safety of bedinvetmab as a long-term therapeutic option, particularly in patients where NSAID use is limited or contraindicated.

From a practical standpoint, these results support the incorporation of bedinvetmab into multimodal OA management strategies in obese dogs, particularly when mobility impairment and chronic pain limit the effectiveness of exercise-based weight management. The observed improvements in activity and QoL also suggest potential indirect benefits for owner engagement and adherence to long-term management plans, reinforcing its relevance within a One Health framework.

A key strength of this study lies in its rigorous randomized controlled design, combined with the integration of both objective (accelerometer-derived activity) and subjective (CBPI, QoL) outcome measures, providing a comprehensive assessment of treatment efficacy. Furthermore, the focus on obese dogs addresses a clinically important yet underrepresented population in anti-NGF research.

In conclusion, bedinvetmab represents an effective and well-tolerated therapeutic option for managing OA in obese dogs, offering significant improvements in pain, mobility, and QoL while contributing to favorable body composition outcomes. These findings support its role as a valuable component of long-term OA management and provide a foundation for future studies exploring its integration with dietary and rehabilitation interventions.

## DATA AVAILABILITY

The datasets generated and/or analyzed during the current study are available from the corresponding author upon reasonable request.

## AUTHORS’ CONTRIBUTIONS

AP: Methodology, data collection, and data analysis. SP: Conception and design of the study. CT: Imaging analysis. TC: Formal analysis and investigation. TT: Materials preparation and data collection. KS: Methodology, study conception, and supervision. All authors have read and approved the final version of the manuscript.
